# Establishing next-generation reference intervals for pro-gastrin-releasing peptide using a dynamic modeling approach

**DOI:** 10.1186/s12967-025-07014-z

**Published:** 2025-09-02

**Authors:** Dong Zhu, Haibin Zhao, Weicheng Zhang, Xiuying Zhao

**Affiliations:** 1 Department of Laboratory Medicine, Beijing Tsinghua Changgung Hospital, School of Clinical Medicine, Tsinghua Medicine, Tsinghua University, No. 168 Litang Road, Changping District, Beijing, 102218 China; 2 Medical College, Dalian University, Dalian, 116622 China

**Keywords:** Pro-gastrin-releasing peptide, Reference intervals, Next-generation reference intervals, Generalized additive models for location, Scale, Shape, Age-related variation

## Abstract

**Background:**

Serum pro-gastrin-releasing peptide (ProGRP) levels significantly vary with age. However, conventional partitioned reference intervals (RIs) fail to reflect the continuous and subtle physiological changes associated with aging. To address this limitation, we investigated the age- and sex-specific dynamics of ProGRP levels and innovatively developed next-generation RIs for adults and elderly individuals in North China.

**Methods:**

A total of 4136 rigorously screened individuals (aged 20–80 years) were analyzed. The effects of age and sex on ProGRP levels were assessed using Mann–Whitney U tests, Spearman’s correlation, and Kruskal–Wallis tests. The Harris–Boyd method was used to evaluate the necessity of sex or age partitioning. Age-partitioned RIs were established using a nonparametric method. Next-generation RI models were developed using generalized additive models for location, scale, and shape, with age-related dynamics visualized. The clinical applicability of both RI types was evaluated by comparing upper reference limit flagging rates across age subgroups; rates closer to the theoretical 2.5% were considered indicative of superior RI accuracy.

**Results:**

Sex had a minimal and clinically insignificant effect on ProGRP levels. In contrast, age had a significant effect: concentrations remained stable until approximately 40 years of age, followed by progressive increases. The partitioned RIs were defined as 0–52.77 pg/mL (20–49 years) and 0–63.68 pg/mL (≥ 50 years). Next-generation RIs were quantified and visualized. Compared with partitioned RIs, next-generation RIs yielded reference limit flagging rates more closely aligned with the theoretical 2.5% across most age groups and demonstrated significantly greater stability (1.83%–3.74% vs. 1.07%–7.10%).

**Conclusions:**

Compared with partitioned RIs, next-generation RIs for ProGRP improve the accuracy of laboratory result interpretation. With advances in laboratory informatics, broader clinical implementation of next-generation RIs is anticipated.

## Background

Pro-gastrin-releasing peptide (ProGRP) is the precursor of the gastrin-releasing peptide, a neuropeptide secreted by cells of neural and endocrine origin [1]. Extensive research has identified ProGRP as a critical biomarker for the diagnosis, treatment monitoring, and response evaluation of small cell lung cancer [2–4]. Furthermore, elevated ProGRP levels can occur in benign conditions, including chronic kidney disease and respiratory diseases [5, 6].

Reference intervals (RIs) are essential for the accurate interpretation of biomarker test results. Overly broad RIs may delay early diagnosis, whereas excessively narrow RIs may mislabel physiologically elevated results as pathological, causing undue patient anxiety and unnecessary medical interventions, particularly in the context of tumor marker testing [7, 8].

Studies have suggested that ProGRP levels could be influenced by sex and age [9–11]; however, most laboratories currently adopt manufacturer-provided RIs that are not stratified by these factors, compromising their clinical validity. Although some studies have established age-partitioned ProGRP RIs [10, 12], these static thresholds fail to reflect the continuous nature of age-related physiological changes. Abrupt transitions in RIs at specific age cutoffs may lead to misinterpretation of test results and increase the risk of misdiagnosis [13].

Recently, researchers have begun studying continuous RIs, known as next-generation RIs, which utilize advanced statistical modeling to construct dynamic, age-dependent reference curves rather than static reference limits [13–17]. Building on this foundation, our team previously evaluated four methodological approaches and innovatively developed next-generation RIs for neuron-specific enolase (NSE) [18]. These next-generation RIs overcome the limitations of conventional partitioned RIs by eliminating artificial age thresholds, thereby reducing the risk of false positives and negatives and potentially enhancing clinical decision-making [13, 17]. However, next-generation RIs for ProGRP have not been reported to date.

In this study, we enrolled 4136 rigorously screened healthy individuals from North China. Partitioned RIs for ProGRP were derived using a nonparametric method. Next-generation RIs were developed using generalized additive models for location, scale, and shape (GAMLSS). A comparative analysis of both approaches was conducted, demonstrating the advantages of next-generation RIs in providing a more precise, age-specific interpretation of ProGRP levels.

## Methods

### Study population

**Fig. 1:**
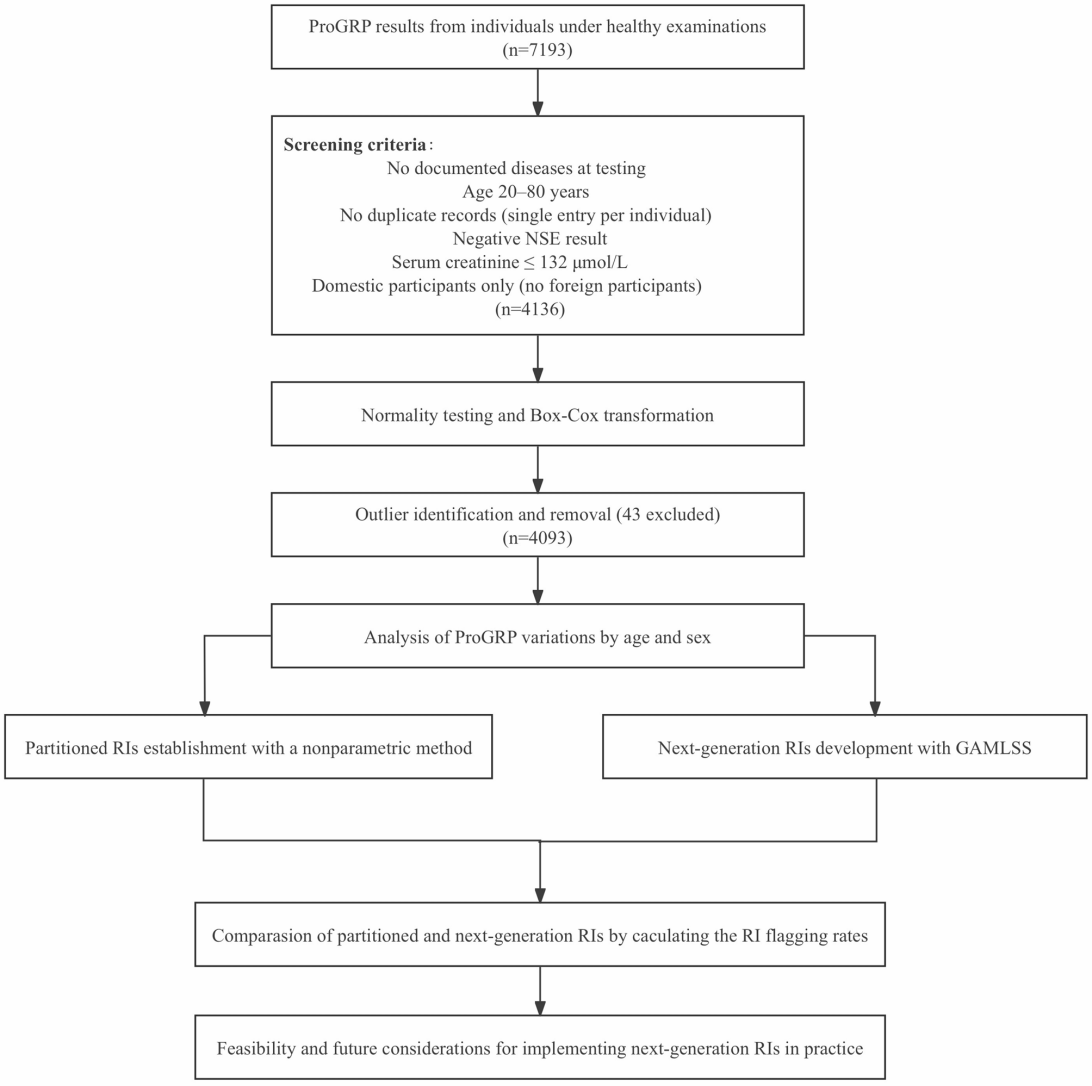
Screening procedures and flowchart of this study. ProGRP, pro-gastrin-releasing peptide; NSE, neuron-specific enolase; RIs, reference intervals; GAMLSS, generalized additive models for location, scale, and shape

This study initially included 7193 serum ProGRP test results from individuals who underwent routine health examinations at the Health Examination Center of Beijing Tsinghua Changgung Hospital between January 2020 and February 2024. Participants were eligible if they were aged 20–80 years and had no documented history of major diseases—such as cancer, chronic kidney disease, or neuroendocrine disorders—at the time of blood collection. As NSE provides comparable diagnostic value to ProGRP, only those with NSE-negative results (defined as < 16.4 µg/L for males, < 14.47 µg/L for females under 50, and < 17.25 µg/L for females ≥ 50) were included [18]. Participants with creatinine values > 133 µmol/L were excluded to avoid potentially falsely elevated ProGRP values [19]. Duplicate records from the same individual and data from non-Chinese residents were also removed to ensure population homogeneity. After applying these criteria, 4136 participants (57.5%) were retained for analysis. The screening process and study workflow are illustrated in Fig. 1.

### Biomarker detection

Serum ProGRP, NSE, and creatinine levels were measured in the clinical laboratory of Beijing Tsinghua Changgung Hospital, which is ISO 15189 accredited. ProGRP was measured via a chemiluminescence immunoassay using an Alinity i analyzer (Abbott, Abbott Park, IL, USA) with manufacturer-supplied reagents, calibrators, and quality control materials. For quality control at low, medium, and high concentrations, the coefficient of variation was under 7.5%. NSE was measured using a Cobas e801 analyzer (Roche Diagnostics, Basel, Switzerland) via an electrochemiluminescence immunoassay with original NSE test kits and calibrators. Quality control was performed using Lyphochek Tumor Marker Plus (Bio-Rad, Hercules, CA, USA) at two concentrations, achieving a coefficient of variation under 5%. Creatinine was measured on a Cobas c702 analyzer (Roche Diagnostics, Basel, Switzerland) using the enzymatic method with original kits and calibrators. For quality control, the Liquid Assay Multiqual (Bio-Rad, Hercules, CA, USA) was used at three concentrations, with the coefficient of variation maintained below 3%.

### Statistical analysis

#### Data preprocessing

Data normality was evaluated using the Kolmogorov‒Smirnov test. When necessary, a Box‒Cox transformation was applied to improve the normality of the data. The outliers were identified using the Tukey method.

#### Sex and age effects on ProGRP

Sex-based differences in ProGRP levels were analyzed with the Mann‒Whitney U test. The correlation between age and the ProGRP level was assessed using Spearman’s correlation analysis. The participants were subsequently stratified into six age groups (20–29, 30–39, 40–49, 50–59, 60–69, and 70–80 years), and differences in ProGRP levels among these groups were analyzed using the Kruskal–Wallis test, followed by Dunn’s post hoc test for pairwise comparisons.

#### Establishment of partitioned RIs

The necessity of partitioning RIs by sex or age was evaluated using the Harris and Boyd methods [20]. Specifically, grouping by sex or age was evaluated by comparing the calculated z value with the critical threshold z⁎, with partitioning deemed necessary when the observed z value exceeded z⁎.

In accordance with the recommendations of the Clinical and Laboratory Standards Institute’s EP28-A3c document, a nonparametric method was employed to establish partitioned RIs for ProGRP [11]. Given the clinical relevance of ProGRP, one-sided RIs (upper limit only) were established, with the 97.5th percentile used as the upper reference limit (URL).

#### Development of the next-generation RI model

GAMLSS was applied to describe the age-dependent dynamics of the ProGRP values and to construct continuous 97.5th percentile curves for next-generation RIs. The Box–Cox power exponential (BCPE) distribution was selected to address skewness and kurtosis in the data. Cubic splines were used to smooth the relationship between age and ProGRP, with an optimal degree of freedom set at 5. The predicted 50th percentile curve represents the central tendency of the ProGRP values across ages, whereas the 97.5th percentile curve defines the upper boundary of the continuous RIs.

#### Comparison of partitioned and next-generation RIs

The reference limit flagging rate is the percentage of test results that exceed the URL or fall below the lower reference limit (LRL). As RIs are defined to encompass the central 95% of values in a reference population, an observed URL or LRL flagging rate approaching the theoretical value of 2.5% reflects greater accuracy of the RIs in the corresponding age population. We evaluated the clinical applicability of partitioned versus next-generation RIs by comparing observed URL flagging rates across six age groups (20–29, 30–39, 40–49, 50–59, 60–69, and 70–80 years).

### Software

All the statistical analyses were conducted using R (version 4.3.0). The GAMLSS model was constructed using the “gamlss” package in R. The significance level for the statistical tests was set at 0.05.

## Results

### Data processing and participant characteristics

A total of 4136 individuals were included in the study after rigorous screening, and 43 statistical outliers were identified and excluded from further analysis. Descriptive statistics for age, sex, and ProGRP levels are summarized in Table 1.

**Table 1:** Age, sex, and ProGRP distributions of the participants

Age groups	Male				Female		
	Subjects (n)	Median	IQR		Subjects (n)	Median	IQR
		(pg/mL)				(pg/mL)	
20–29	240	30.87	10.17		268	31.25	10.52
30–39	698	30.59	10.32		569	31.59	10.93
40–49	635	33.51	12.48		419	31.9	11.28
50–59	469	35.14	11.67		276	34.69	13.28
60–69	210	36.88	15.38		200	36.4	16.96
70–80	54	39.61	13.75		55	43.25	18.01
Total	2306	33.08	12.25		1787	32.62	11.82

ProGRP, pro-gastrin-releasing peptide; IQR, interquartile range

### Sex-based ProGRP variations and RI stratification

**Fig. 2:**
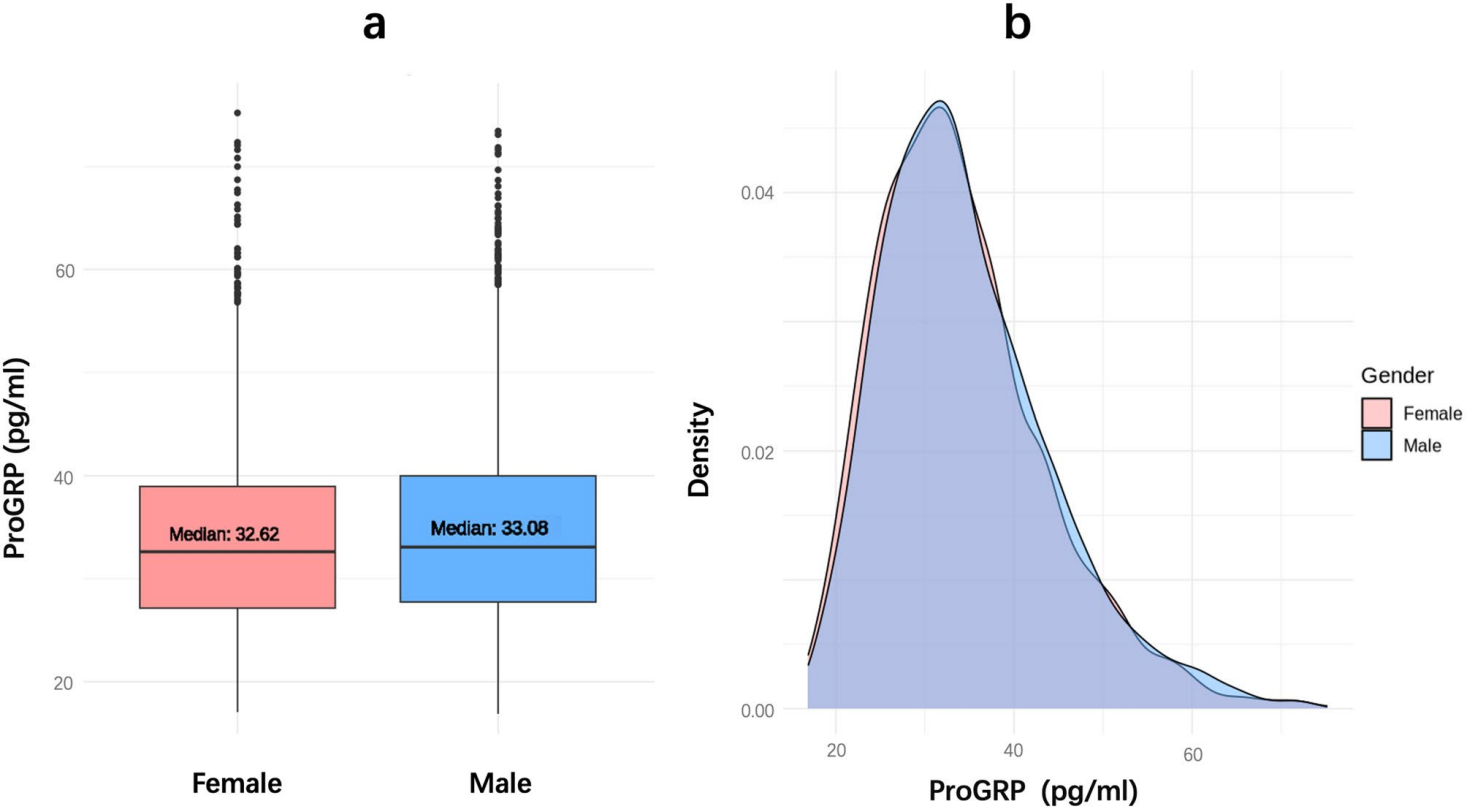
ProGRP levels by sex boxplot and density distribution. **a** presents a boxplot with median ProGRP levels of 32.62 pg/mL for females and 33.08 pg/mL for males. Although a significant difference was observed, the effect size was minimal, with a Cohen’s d of 0.066. The overlapping distributions in the density plot (**b**) further emphasize the negligible variation. ProGRP, pro-gastrin-releasing peptide

The Mann‒Whitney U test indicated that ProGRP levels were significantly higher in males than in females (*p* = 0.038). However, the median values were very close (32.62 pg/mL for females and 33.08 pg/mL for males), with a Cohen’s d of 0.066, indicating a trivial effect size (below the 0.2 threshold). Thus, despite statistical significance, the sex-related variation in ProGRP is unlikely to be clinically meaningful [21]. The overlapping distributions observed in the density plot further confirm the negligible magnitude of the difference (Fig. 2). The necessity for sex-based RI establishment was further evaluated using the Harris and Boyd method [20]. The calculated z value of 2.091 was much smaller than the critical z* value of 12.374, indicating that sex-based stratification of RIs is not warranted.

### Age-based ProGRP dynamics and RI stratification

**Fig. 3:**
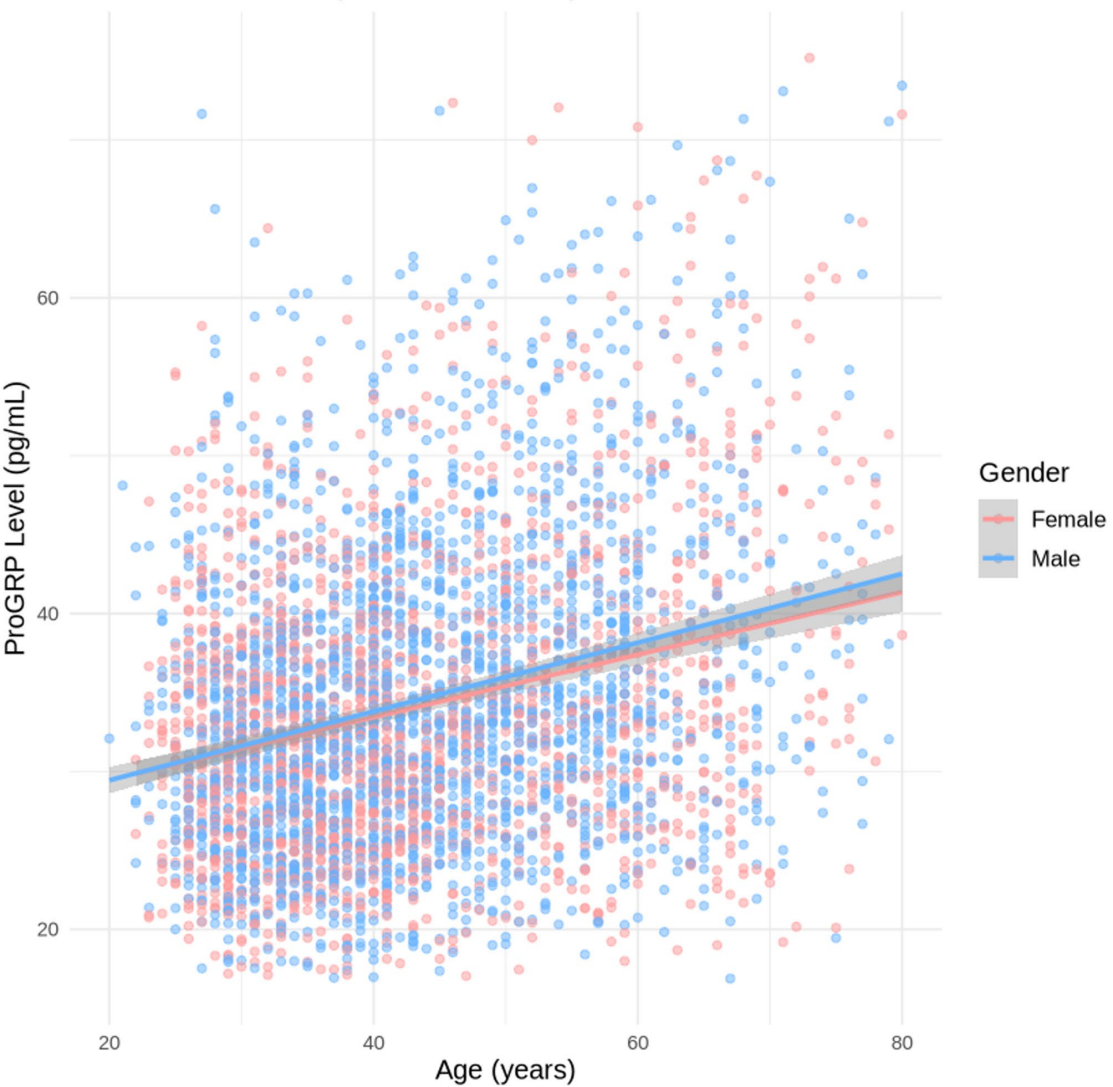
Correlation analysis between age and ProGRP levels. This graph illustrates the relationship between age and ProGRP levels, with blue and pink points representing male and female data, respectively. In both sexes, ProGRP levels increased with age, following a similar dynamic pattern. ProGRP, pro-gastrin-releasing peptide

Spearman’s correlation analysis revealed a positive correlation between ProGRP levels and age (r = 0.24, *p* < 0.001), with r = 0.26 (*p* < 0.001) in males and r = 0.21 (*p* < 0.001) in females (Fig. 3). In both sexes, ProGRP levels increased with age, following a similar dynamic pattern. The Kruskal‒Wallis test confirmed significant differences in ProGRP levels across the six age groups (*p* < 0.001). Pairwise comparisons via Dunn’s test provided further details (Table 2). Considering both the statistical significance and the clinical utility of ProGRP interpretation, we established RIs stratified by age: 20–49 years and ≥ 50 years. This stratification was validated using the Harris and Boyd method, where the calculated z statistic (14.617) exceeded the critical value (z* = 12.374), statistically justifying age-specific RIs.

**Table 2:** Pairwise comparisons of ProGRP levels among different age groups

Age group	z Value	*p* Value
(20‐29) vs. (30–39)	0.521	1.0
(20‐29) vs. (40–49)	-4.622	** < 0.001**
(20‐29) vs. (50–59)	-8.532	** < 0.001**
(20‐29) vs. (60–69)	-9.095	** < 0.001**
(20‐29) vs. (70–80)	-8.336	** < 0.001**
(30‐39) vs. (40–49)	-6.644	** < 0.001**
(30‐39) vs. (50–59)	-11.226	** < 0.001**
(30‐39) vs. (60–69)	-11.109	** < 0.001**
(30‐39) vs. (70–80)	-9.09	** < 0.001**
(40‐49) vs. (50–59)	-5.041	** < 0.001**
(40‐49) vs. (60–69)	-6.085	** < 0.001**
(40‐49) vs. (70–80)	-6.265	** < 0.001**
(50‐59) vs. (60–69)	-1.836	0.995
(50‐59) vs. (70–80)	-3.794	**0.002**
(60‐69) vs. (70–80)	-2.562	0.156

Statistically significant differences are highlighted in bold (*p* < 0.05). ProGRP, pro-gastrin-releasing peptide

### Age-partitioned RI establishment

The age-specific ProGRP RIs determined via the partitioned method are as follows:

- 20–49 years: 0–52.77 pg/mL (90% CI for URL: 52.04–53.88)
- ≥50 years: 0–63.68 pg/mL (90% CI for URL: 61.40–64.95)

### Next-generation RIs for ProGRP

Using GAMLSS, we model the age-related dynamics of ProGRP by fitting a continuous median curve and generating continuous 97.5th percentiles, which serve as the URLs of the next-generation RIs.

**Fig. 4:**
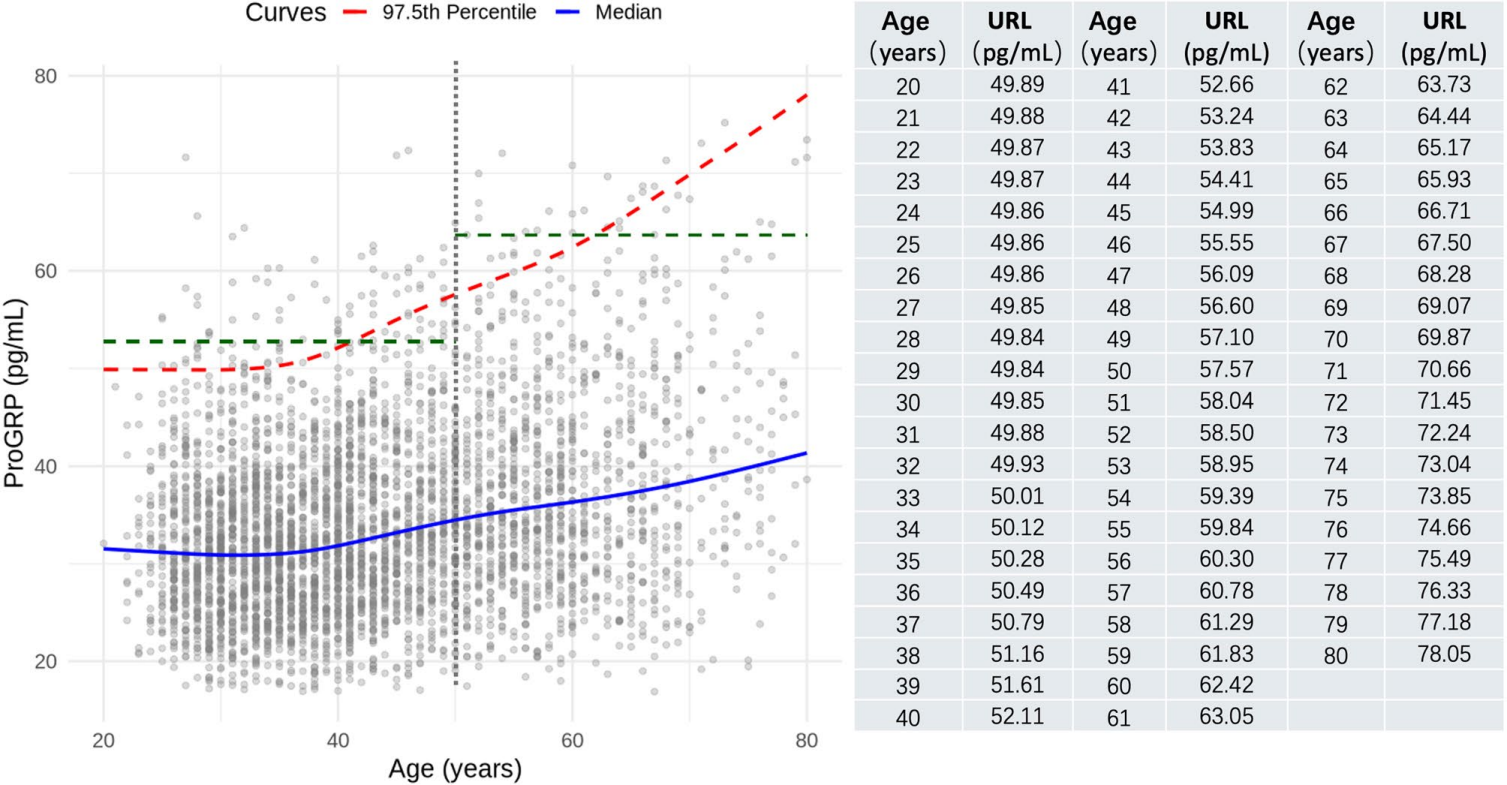
Dynamic age-related trends and next-generation RIs for ProGRP. The green dashed lines indicate the age-partitioned URLs for ProGRP established in this study. The blue fitted line, derived from GAMLSS, illustrates the dynamic age-related trend of ProGRP levels. The red dashed line represents the continuous 97.5th percentile, indicating the upper limit of the next-generation RIs. Additional details are provided on the right. ProGRP, pro-gastrin-releasing peptide; RIs, reference intervals; GAMLSS, generalized additive models for location, scale, and shape; URL, upper reference limit

Overall, the ProGRP levels remained stable up to age 40, with median values fluctuating minimally between 30.88 and 31.62 pg/mL and corresponding 97.5th percentiles ranging from 49.84 to 51.61 pg/mL. After age 40, a noticeable increase in ProGRP levels was observed. The median concentration rose from 31.85 pg/mL at age 40 to 41.36 pg/mL at age 80, representing a 29.9% increase. In parallel, the continuous 97.5th percentile increased from 52.11 pg/mL at age 40 to 78.05 pg/mL at age 80—a 49.8% increase. The nonlinear age-related trajectory and the next-generation RIs for ProGRP are illustrated in detail in Fig. 4.

### Comparison of partitioned and next-generation RIs

To evaluate the clinical applicability of partitioned and next-generation RIs for ProGRP, we compared the observed URL flagging rates across six age groups. As shown in Table 3, both RI types yielded near-ideal URL flagging rates in the overall population. However, in five of the six age subgroups, next-generation RIs yielded flagging rates more closely aligned with the theoretical 2.5%, indicating substantially better concordance with the underlying distribution. Moreover, next-generation RIs demonstrated greater stability, with flagging rates ranging from 1.83% to 3.74%, than the broader variability observed with partitioned RIs (1.07%–7.10%).

**Table 3:** Upper reference limit flagging rates for partitioned and next-generation RIs across different age groups

Age groups	Subjects (n)	Partitioned RIs				Next-generation RIs	
		Flagged subjects (n)	Flagging rate			Flagged subjects (n)	Flagging rate
20–29	508	10	1.97%			19	3.74%
30–39	1267	16	1.26%			31	2.45%
40–49	1054	45	4.27%			33	3.13%
50–59	745	8	1.07%			16	2.15%
60–69	410	16	3.90%			11	2.68%
70–80	109	8	7.30%			2	1.83%
Total	4093	103	2.52%			112	2.74%

RIs, reference intervals

## Discussion

ProGRP levels exhibit significant age-dependent variation; however, conventional static RIs fail to reflect gradual dynamics, increasing the risk of misinterpretation and diagnostic error. In this study, we established both age-partitioned and next-generation RIs for ProGRP in adults and elderly individuals in North China. Through a comparative analysis of the two approaches, we detail the advantages of next-generation RIs in clinical practice. To the best of our knowledge, this study is the first to develop next-generation RIs for ProGRP.

The age-partitioned RIs established in this study provide several advantages over the manufacturer’s RI for ProGRP (0–63 pg/mL), which lacks age stratification and was derived from 194 individuals of unspecified demographics. First, our RIs are based on a substantially larger dataset (4136 individuals), enhancing statistical robustness. Second, our RIs provide better geographical specificity. The reference population in this study was drawn from a tertiary hospital in Beijing, a major hub in North China, and included both long-term residents and migrants, ensuring demographic diversity representative of northern Chinese populations [22]. Third, partitioned RIs offer improved age-specific accuracy. Although the manufacturer’s RI closely aligns with our RI for individuals aged ≥ 50 years, it is excessively broad for young and middle-aged adults, which risks false-negative interpretations in these populations.

Although age-partitioned RIs offer advantages over nonstratified RIs, next-generation RIs for ProGRP evidently more accurately reflect gradual age-related dynamics and exhibit better clinical applicability. In Fig. 4, a visual comparison between the two approaches highlights these differences. Partitioned RIs exhibit abrupt transitions that fail to reflect gradual changes in biomarker concentrations, potentially leading to misclassification. In this study, the URL in the partitioned RIs exhibited an abrupt 20.6% increase between ages 49 and 50. In contrast, the next-generation RIs demonstrated a modest 0.8% increase in the same age range, more accurately capturing the subtle physiological trajectory with age. Moreover, the inherent limitation of static RIs is particularly pronounced in populations with high age-related ProGRP variability, such as the elderly in this study. The 7.30% URL flagging rate observed in the 70–80 age group using partitioned RIs clearly represents an unacceptable proportion. By definition, RIs encompass the central 95% of test values in a healthy population; thus, an observed flagging rate (URL or LRL) close to the ideal 2.5% suggests better conformity with the true distribution and higher RI accuracy. In this study, URL flagging rates across age groups were significantly more stable and mostly closer to the ideal 2.5% when using next-generation RIs, confirming their superior age-specific accuracy and better clinical applicability across diverse populations.

Several studies have investigated the influence of sex on ProGRP levels, with inconsistent results. One study conducted in Korea reported that sex had a significant influence on ProGRP levels and recommended the use of sex-specific RIs [10]. In contrast, two other studies reported no significant sex effect [9, 12], and such discrepancies are likely attributable to differences in geographic populations, sample sizes, or statistical power. In this study, although we observed a statistically significant difference in ProGRP levels between sexes, which is consistent with the findings of Nah et al. [10], we did not recommend sex-specific RIs on the basis solely of between-group comparisons, as they did. Instead, we adopted the more rigorous approach proposed by Harris and Boyd [20] and concluded that sex-specific RIs were not necessary. This is because the large sample sizes used in between-group comparisons can easily produce statistically significant differences even when the actual effect size is minimal and clinically negligible [23]. Therefore, a more evidence-based and methodologically sound RI stratification approach, such as the Harris and Boyd method, could be more scientifically valid.

With respect to the age effect, all the referenced studies demonstrated a strong correlation between ProGRP levels and age, corroborating our findings [9, 10, 12] and underscoring the necessity of age-specific RIs for ProGRP in clinical practice.

The EP28-A3c guidelines delineate two sampling methods for RI establishment: 1) the direct method involving a priori or a posteriori recruitment of reference individuals under rigorous screening criteria and 2) the indirect method utilizing retrospective laboratory data, such as those from outpatient clinics or medical examination centers [11]. Although the direct method is generally considered the preferred approach, its operational complexity and resource intensity have led to the indirect method becoming an increasingly recommended alternative for establishing RIs [24–27].

Previous studies have shown that, with sufficiently large sample sizes, appropriate outlier exclusion strategies, and robust statistical approaches, the inclusion of certain “abnormal” values in datasets—such as those derived from outpatient populations—does not compromise the reliability of RI estimation using the indirect method [28–30]. In our study, to further enhance the validity of the derived RIs, we used data exclusively from individuals undergoing routine health examinations rather than from clinical patients. Stringent screening criteria were applied, including the exclusion of individuals with known or suspected ProGRP-related conditions—such as cancer, chronic kidney disease, or neuroendocrine disorders—as well as those with abnormal levels of relevant biomarkers such as NSE and creatinine. These screening measures were adopted to further strengthen the credibility of the RIs established in this study.

Moreover, the direct method tends to result in limited sample sizes, which constrains the feasibility of multidimensional stratification [26, 27]. In contrast, our study utilized a large-scale dataset, improving the statistical robustness of partitioned RIs and enhancing model-fitting performance for next-generation RIs [31].

Although the RIs established in this study offer advantages over manufacturer-provided RIs, both analytical compatibility and population applicability must be verified prior to clinical implementation [11]. One study reported significant differences in ProGRP quantification between two widely used immunoassay platforms: Elecsys (Roche, Cobas e602) and Architect (Abbott, i2000SR) [32]. If test results are not analytically comparable, transferring RIs across platforms is not justified [11, 33]. Therefore, analytical compatibility with the assay platform used in this study must be confirmed before applying these RIs. Additionally, regional differences in biomarker levels—due to genetic, lifestyle, dietary, or environmental factors—may limit the generalizability of RIs [34, 35]. As our RIs were established on the basis of a northern Chinese population, their generalizability to other populations remains uncertain. Therefore, caution is advised when applying these RIs to other regions or countries. Verification of RIs prior to clinical implementation is essential to ensure their applicability in specific testing contexts [11].

While next-generation RIs are not yet commonly implemented in clinical laboratories, owing primarily to challenges related to methodological complexity and technological constraints, they hold considerable potential for clinical utility. Several techniques, such as fractional polynomial regression, nonparametric quantile regression, the LMS (lambda-mu-sigma) model, and GAMLSS, have been explored as potential methods for developing continuous RIs [15, 36–38]. We selected GAMLSS given its superior ability to fit complex curves and minimize edge effects, which makes GAMLSS an ideal tool for modeling continuous RIs [18, 36, 39]. A feasible strategy for implementing next-generation RIs in clinical practice is to integrate the established models into laboratory middleware or the laboratory information system (LIS). This would enable patient-specific RI calculations on the basis of age and support dynamic flagging of test results that fall outside the individualized RIs.

This study has several limitations. As a real-world study, its internal validity may not be as robust as that of a clinical study. However, we enhanced its reliability by using a large dataset, applying stringent inclusion criteria, and employing rigorous statistical methods. Additionally, ProGRP concentrations were measured using the Abbott Alinity i analyzer with a chemiluminescent immunoassay. The established RIs may not be directly transferable to other analytical platforms or assay methodologies. The applicability of these RIs across multiple testing platforms remains to be further evaluated through analytical comparability verification. Third, the study population was derived from a single center in North China and included both long-term residents and migrants. Regional genetic, dietary, and environmental factors may limit the generalizability of these reference intervals to populations in other geographic regions. Verification of these RIs in the target population is essential before clinical implementation. Fourth, the next-generation RIs demonstrated superior age-specific accuracy in healthy individuals compared with conventional RIs. However, their diagnostic sensitivity and specificity—relative to fixed thresholds—have not been assessed in disease-specific populations. Further validation in patients with small cell lung cancer or other neuroendocrine tumors is warranted to determine their clinical utility. Finally, despite the demonstrated advantages of next-generation RIs, their adoption in most clinical laboratories remains challenging at present owing to methodological complexity and technological constraints.

## Conclusions

This study investigated age- and sex-related variations and innovatively developed age-dependent next-generation RIs for ProGRP in adult and elderly populations in North China. Compared with conventional static RIs, next-generation RIs more precisely characterize the distribution of ProGRP in healthy populations, thereby improving the accuracy of laboratory result interpretation. Although challenges remain in their clinical implementation, increasing recognition of next-generation RIs and advancements in information technology are expected to facilitate their adoption in clinical practice.

## Data Availability

The datasets used and/or analyzed during the current study are available from the corresponding author upon reasonable request.

## Abbreviations

ProGRP: Pro-gastrin-releasing peptide
NSE: Neuron-specific enolase
RIs: Reference intervals
GAMLSS: Generalized additive models for location, scale, and shape
URL: Upper reference limit
LRL: Lower reference limit
LIS: Laboratory information system
